# Pleth Variability Index or Inferior Vena Cava Collapsibility Index? Prospective Observational Study in Volume Control and Follow-Up Acute Kidney Injury

**DOI:** 10.3390/medicina61101868

**Published:** 2025-10-17

**Authors:** Ecem Ermete Güler, Ejder Saylav Bora, Hüseyin Acar, Süleyman Kırık, Burak Acar, Şakir Hakan Aksu

**Affiliations:** 1 Department of Emergency Medicine, Izmir Ataturk Training and Researcing Hospital, 35360 Izmir, Türkiye; kiriksuleyman2107@outlook.com; 2 Department of Emergency Medicine, Faculty of Medicine, Izmir Katip Celebi University, 35620 Izmir, Türkiye; saylavbora@hotmail.com (E.S.B.); dracar@hotmail.com (H.A.); 3 Department of Emergency Medicine, Adıyaman University Training and Researcing Hospital, 02040 Adıyaman, Türkiye; burakacar7493@gmail.com; 4 Department of Emergency Medicine, Izmir City Hospital, 35170 Izmir, Türkiye; aksu.sakirhakan@gmail.com

**Keywords:** Pleth Variability Index, acute kidney injury, fluid responsiveness, Inferior Vena Cava Collapsibility Index

## Abstract

*Background and Objective*: Acute kidney injury (AKI) is a serious condition requiring prompt fluid resuscitation, yet both under- and over-treatment carry risks. Accurate volume assessment is essential, especially in emergency settings. The Inferior Vena Cava Collapsibility Index (IVCCI) is commonly used but has limitations. The Pleth Variability Index (PVI) offers a non-invasive alternative, though its role in AKI remains unclear. To compare the efficacy of the Pleth Variability Index (PVI) and Inferior Vena Cava Collapsibility Index (IVCCI) in assessing fluid responsiveness and predicting in-hospital mortality in patients with acute kidney injury. *Materials and Methods*: This prospective observational study enrolled 50 adult AKI patients presenting to a tertiary emergency department. All patients received sequential fluid resuscitation with 1000 mL and 2000 mL of isotonic saline. PVI, IVCCI, mean arterial pressure (MAP), peripheral oxygen saturation (SpO_2_, perfusion index (PI), and shock index (SI) were recorded at baseline and after each fluid bolus. Changes in these parameters were analyzed to assess their utility in fluid responsiveness. Additionally, the prognostic value of baseline PVI for in-hospital mortality was investigated. *Results*: PVI demonstrated a significant and dose-responsive decrease following fluid administration, outperforming IVCCI, MAP, PI, SpO_2_, and SI in sensitivity (*p* < 0.001). Baseline PVI values were significantly associated with mortality (AUC: 0.821, *p* < 0.001), whereas post-resuscitation PVI values showed no prognostic significance. IVCCI and PI showed comparable reliability but were less sensitive to incremental volume changes. *Conclusions*: PVI is a sensitive, non-invasive marker of fluid responsiveness in non-intubated AKI patients and may also serve as an early prognostic indicator. Its use in emergency departments could support fluid management decisions, but further large-scale, multicenter studies are needed to validate these findings.

## 1. Introduction

Acute kidney injury (AKI) is a rapid deterioration of renal function, frequently observed in hospitalized and critically ill (such as sepsis and post-operative) individuals [1]. It is a complicated, multifaceted illness with considerable short- and long-term health ramifications, including an elevated risk of chronic renal disease and mortality. AKI is defined by a swift elevation in blood creatinine levels, diminished urine output, or both, indicating an abrupt decline in the kidney’s excretory capacity [2,3]. Diagnosis is based on alterations in nitrogen metabolism byproducts (urea, creatinine) or oliguria [4,5]. Timely identification, prophylaxis, and supportive care are crucial for enhancing outcomes, as no definitive treatments consistently reverse established AKI.

Fluid resuscitation is a crucial component of acute kidney injury therapy; nevertheless, current studies underscore the intricacies and possible hazards associated with both under- and over-resuscitation [6]. The kind, volume, and timing of fluids, together with methods for fluid evacuation, are widely acknowledged as key elements affecting patient outcomes [7]. Adequate fluid resuscitation is essential in the management of AKI, and many patients may require excessive amounts of fluid depending on the hemodynamic response [8,9]. Replacement of fluid deficit is the most important step of treatment. Therefore, it is important to assess the fluid volume of patients and manage it appropriately.

The Inferior Vena Cava Collapsibility Index (IVCCI) is one of the most often utilized techniques in clinical practice to evaluate hydration status [10]. This index gives information regarding intravascular volume status and is computed using the change in inferior vena cava (IVC) diameter with breathing. Numerous elements, including operator dependence, patient position, abdominal pressure, variations in technological application, and the patient’s respiratory cooperation, can influence the use of this procedure [11]. According to research published in the literature [12,13], not all patient groups can be reliably predicted to respond fluidly by the IVCCI. The method’s poor accuracy, particularly in individuals who breathe on their own, prevents it from being widely used [14]. Therefore, there is a growing need for alternative measurement methods that are less operator-dependent, standardized, and non-invasive.

The Pleth Variability Index (PVI) quantifies respiratory fluctuations in the amplitude of the pulse oximeter waveform, indicating alterations in intravascular volume and fluid responsiveness, especially in patients undergoing mechanical ventilation [15,16]. By assessing changes brought on by breathing, PVI gives information on intravascular volume status and can help direct fluid therapy [17]. PVI may, therefore, be utilized as a predictive indicator for AKI patients’ fluid control and circulatory sufficiency. However, data on the prognostic value of PVI in AKI patients are limited.

AKI is a disease with high morbidity and mortality requiring aggressive intravenous hydration therapy. The amount and adequacy of fluid intake in the first hours of these patients whose initial treatment is organized in the emergency department is important for the prognosis of the disease.

In this study, we aimed to demonstrate the effectiveness of the PVI and IVCCI as easily accessible (low cost and low risk of infection), non-invasive, and rapid measurement methods in the evaluation of response to treatment and their advantages over each other in pre-renal acute kidney-disease-induced AKI.

## 2. Materials and Methods

### 2.1. Study Design and Setting

This prospective observational study was conducted in the Emergency Department of Izmir Ataturk Training and Research Hospital, a tertiary care center with a high patient volume. The aim was to investigate the clinical utility of two non-invasive dynamic monitoring parameters—the Pleth Variability Index (PVI) and the Inferior Vena Cava Collapsibility Index (IVCCI)—in evaluating fluid responsiveness among adult patients with pre-renal acute kidney-disease-induced AKI. The study period extended from 1 January 2024 to 30 June 2024. Consecutive patients aged 18 years or older, who were diagnosed with AKI at presentation and fulfilled the inclusion criteria, were enrolled. All participants underwent standardized fluid resuscitation with 1000 mL followed by 2000 mL isotonic saline. Hemodynamic variables, including PVI, IVCCI, mean arterial pressure (MAP), perfusion index (PI), shock index (SI), and peripheral oxygen saturation (SpO_2_), were recorded at baseline and after each fluid bolus. To minimize inter-observer variability, IVCCI measurements were performed by two experienced emergency physicians trained in point-of-care ultrasonography. PVI monitoring was conducted continuously using a pulse oximetry device equipped with integrated pleth variability software. The study was designed to enable a controlled comparison of PVI and IVCCI in spontaneously breathing, non-intubated AKI patients under real-life emergency conditions. The protocol was approved by the Ethics Committee of the University of Health Sciences, Izmir Ataturk Training and Research Hospital (Approval No: 0158; 27 April 2023), in compliance with the Declaration of Helsinki.

### 2.2. Study Population

A total of 50 adult patients with a clinical diagnosis of AKI were enrolled consecutively during the study period. The diagnosis of AKI was established according to Kidney Disease: Improving Global Outcomes (KDIGO) criteria, as follows: an increase in serum creatinine by ≥0.3 mg/dL within 48 h, an increase in serum creatinine to ≥1.5 times baseline within the previous seven days, or urine output < 0.5 mL/kg/h for six hours. Eligible patients were hemodynamically stable, responsive to fluid therapy, had a peripheral oxygen saturation of ≥85%, and were suitable for non-invasive monitoring and bedside ultrasonography. Patients were excluded if they were younger than 18 years, mechanically ventilated, or had significant comorbidities such as severe arrhythmias, intra-abdominal hypertension, ascites, morbid obesity (BMI > 40 kg/m^2^), severe valvular heart disease, or pregnancy. Exclusion also applied to those with poor sonographic windows for IVC assessment, active bleeding, urgent surgical requirements, or those who declined consent. Moreover, post-surgical patients and those transferred from other hospitals were excluded to ensure homogeneity of the study population.

### 2.3. Study Protocol

**Figure 1:**
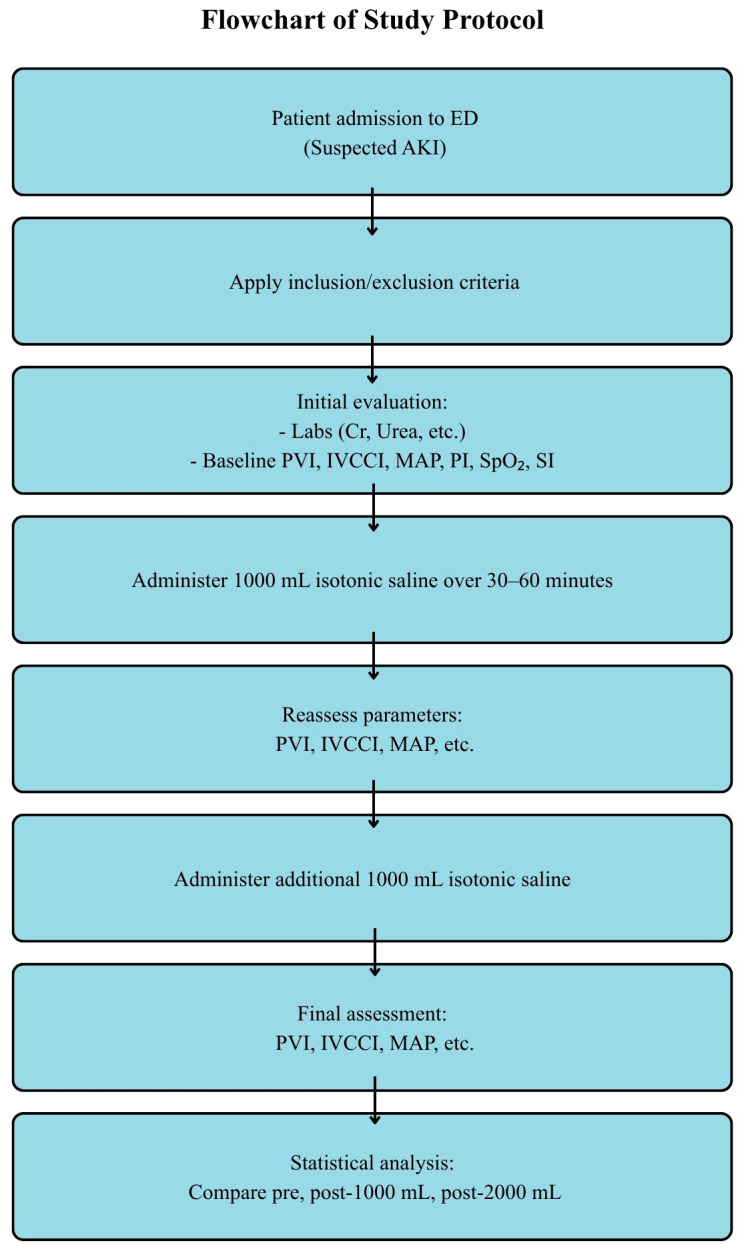
Flowchart of study protocol.

Baseline demographic and clinical data—including age, sex, blood pressure, pulse rate, and laboratory findings (serum creatinine, urea, electrolytes, arterial blood gases)—were recorded upon admission. Hemodynamic and perfusion parameters were measured at the following three time points: (1) before fluid administration, (2) after infusion of 1000 mL isotonic saline over 30–60 min, and (3) after infusion of an additional 1000 mL (total of 2000 mL). At each stage, MAP, SpO_2_, PI, PVI (Masimo Radical-7 pulse oximetry), IVCCI (via subxiphoid long-axis ultrasonography), and SI (calculated as the ratio of heart rate to systolic blood pressure) were assessed. All ultrasound examinations were performed by the same trained emergency physician using a standardized measurement protocol. The workflow chart of patient enrollment and follow-up is illustrated in Figure 1.

IVCCI: We evaluated the Inferior Vena Cava (IVC) Collapsibility Index in all patients using bedside ultrasonography. The measurements were performed with a 3.5–5 MHz phased array transducer in the subxiphoid region. We obtained a longitudinal view of the IVC approximately 2–3 cm caudal to the right atrium. The maximum diameter of the IVC during expiration (IVCmax) and the minimum diameter during inspiration (IVCmin) were measured in M-mode. We calculated the collapsibility index using the following formula: (IVCmax − IVCmin)/IVCmax × 100. All examinations were carried out by experienced emergency physicians who had completed standardized ultrasonography training.

### 2.4. Outcome Measures

#### 2.4.1. Primary Outcome

The primary outcome of the study was the change in PVI and IVCCI values following standardized fluid resuscitation with 1000 mL and 2000 mL isotonic saline in patients diagnosed with AKI.

#### 2.4.2. Secondary Outcomes

Secondary outcomes included the correlation of PVI and IVCCI changes with hemodynamic and perfusion parameters such as MAP, SI, PI, and serum lactate levels. Additionally, the study aimed to determine which index—PVI or IVCCI—more accurately reflects early fluid responsiveness and improvement in tissue perfusion.

### 2.5. Statistical Analysis

All statistical analyses were performed using IBM SPSS Statistics version 27 for Mac. Descriptive statistics are presented as means ± standard deviations for continuous variables and as frequencies and percentages for categorical variables. The Kolmogorov–Smirnov test was used to assess the normality of data distribution.

For comparisons of hemodynamic and perfusion parameters (MAP, SpO_2_, PI, PVI, IVCCI, and SI) measured at three consecutive time points—before fluid administration (T0), after 1000 mL isotonic saline infusion (T1), and after 2000 mL isotonic saline infusion (T2)—a repeated measures analysis was conducted. Because most parameters did not show normal distribution, the Friedman test, a non-parametric equivalent of repeated measures ANOVA, was used to evaluate overall differences among the three time points. When significant differences were detected, pairwise post hoc comparisons were performed using Bonferroni correction to control for multiple testing.

Receiver Operating Characteristic (ROC) curve analysis was applied to assess the predictive performance of baseline PVI values for in-hospital mortality. The area under the curve (AUC) was calculated together with its 95% confidence interval (CI), and the optimal cut-off point was determined using the Youden Index. A two-tailed *p*-value < 0.05 was considered statistically significant.

A sample size of 50 patients was estimated to provide 80% power to detect a clinically meaningful difference in PVI and IVCCI values across repeated measurements, assuming a significance level of 0.05 and based on effect sizes observed in previous pilot studies.

## 3. Results

A total of 50 patients were included in the study, of whom 26 (52%) were female. The mean age was 69.7 ± 15.1 years. Baseline demographic and clinical characteristics are presented in Table 1.

The mean, minimum, and maximum values and standard deviations of hemodynamic and perfusion parameters (MAP, SpO_2_, PI, PVI, IVCCI, and SI) at baseline, after 1000 mL isotonic saline, and after 2000 mL isotonic saline infusion are shown in Table 2.

When repeated measurements of MAP, SPO_2_, PI, PVI, IVCCI, and SI before treatment, after 1000 cc of isotonic infusion, and after 2000 cc of isotonic infusion were compared, a significant difference was observed for each parameter (*p* < 0.001 for each) (Table 3).

Pairwise comparisons of these measurements are summarized in Table 4. MAP significantly increased after both 1000 mL (*p* = 0.013) and 2000 mL (*p* = 0.001) infusion compared to baseline, whereas no further rise was observed between 1000 mL and 2000 mL (*p* = 1.000). SpO_2_ did not change significantly after 1000 mL but was higher after 2000 mL compared with baseline (*p* = 0.002). PI values increased significantly after both 1000 mL (*p* = 0.010) and 2000 mL (*p* < 0.001) compared to baseline, without additional difference between the two infusion points (*p* = 0.329).

Dynamic indices demonstrated distinct trends. PVI values decreased significantly after 1000 mL and further after 2000 mL compared with baseline (both *p* < 0.001), with an additional significant reduction between 1000 mL and 2000 mL (*p* = 0.01). Similarly, IVCCI decreased after both 1000 mL (*p* = 0.006) and 2000 mL (*p* < 0.001) infusion compared to baseline, although there was no difference between the two post-infusion measurements (*p* = 0.056). Shock index values also fell significantly after 1000 mL (*p* = 0.018) and 2000 mL (*p* < 0.001) compared with baseline, but remained similar between the two infusion stages (*p* = 0.363).

When PVI values were analyzed in relation to in-hospital mortality, only baseline PVI (PVI-0) was significantly associated with mortality (*p* < 0.001). In contrast, post-infusion PVI values (PVI-1 and PVI-2) showed no significant relationship (*p* = 0.529 and *p* = 0.914, respectively) (Table 5).

**Figure 2:**
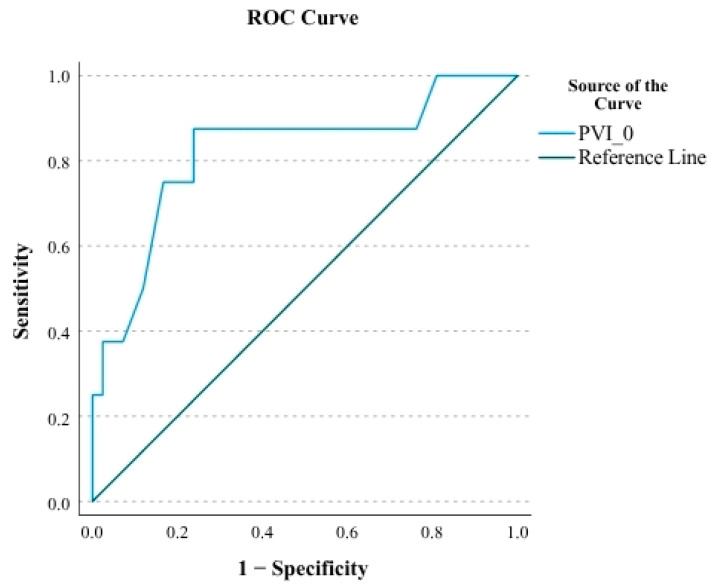
ROC curve showing the success of PVI-0 in mortality prediction.

In ROC analysis, baseline PVI (PVI-0) demonstrated a strong predictive ability for mortality, with an AUC of 0.821 95% CI (Figure 2). Using the Youden index, a cut-off value of 36.5 was identified, yielding a sensitivity of 87.5% and specificity of 76.2%. The corresponding PPV and NPV were 41.2% and 97.0%, respectively (Table 6). These findings suggest that a baseline PVI above 36.5 is a reliable predictor of mortality in AKI patients undergoing fluid resuscitation, primarily due to its high negative predictive value.

## 4. Discussion

Non-invasive monitoring for evaluating fluid resuscitation is feasible and can provide significant insights, particularly when invasive techniques such as central venous catheterization are not available.

In this study, we aimed to evaluate the Pleth Variability Index (PVI), a dynamic parameter mainly used to assess fluid status in mechanically ventilated patients in anesthesia and intensive care units, in addition to known techniques for the evaluation of hydration in acute renal failure patients with urgent fluid needs in the emergency department.

Assessment of MAP and blood volume is essential for directing fluid resuscitation in critically ill and trauma patients. MAP alone may not indicate blood volume status or the need for fluid resuscitation. Research shows that MAP can normalize even after stroke volume and cardiac output return to baseline, resulting in unnecessary fluid administration and fluid overload [18,19,20]. Reduced mean arterial pressure is significantly correlated with heightened risk of AKI and mortality in critically ill and surgical patients. Maintaining an MAP above 65–70 mmHg, and at even higher levels in specific patient groups, seems to offer protective effects [21,22]. In this study, the mean MAP values after 1000 mL and 2000 mL saline infusion were 79 mmHg and 84 mmHg, respectively, showing significant improvement compared with pretreatment status. Although MAP alone is insufficient for fluid replacement assessment, the lack of significant difference between MAP values between 1000 and 2000 cc suggests switching from bolus to slow infusion after 1000 cc to prevent AKI.

Fluid resuscitation following hypovolemic shock typically enhances tissue oxygen delivery and local tissue oxygen saturation, bringing these metrics nearer to baseline levels [23]. In an experimental study conducted by Legrand et al., they observed that fluid resuscitation in rats experiencing hemorrhagic shock fails to enhance renal oxygenation, and blood transfusion does not completely restore it [24]. In this study, we found that SpO_2_ levels alone were inadequate for assessing fluid replacement adequacy; however, a significant increase of 2000 cc in fluid replacement was noted post-treatment. The difficulty of standardization in the emergency department undermines the reliability of this result.

Peripheral perfusion is indicated by the PI, which can be measured continuously and non-invasively as the PVI [25]. A strong negative correlation with arterial lactate levels following fluid resuscitation has been observed. Employing PI for fluid resuscitation in septic shock may decrease lactate levels, reduce the duration to achieve negative fluid balance, and potentially mitigate the risk of fluid overload [26]. De Miranda et al. describe that there was no intrinsic difference in PI between patients with and without sepsis-associated AKI; however, hypoperfusion emerged as a significant predictor of 28-day mortality in the AKI cohort [27]. In this study, when fluid replacement of 1000 and 2000 cc was performed, the fact that meaningful results were obtained based on the patient’s PI upon arrival shows that PI was successful in fluid resuscitation assessment but did not provide us with any clues regarding the adequacy of resuscitation.

The SI, defined as the ratio of heart rate to systolic blood pressure, is increasingly utilized to evaluate severity and inform fluid therapy decisions [28,29]. The application of SI, advanced hemodynamic monitoring, or perfusion indices has the potential to enhance patient outcomes, minimize complications, and prevent unnecessary fluid overload [29]. Li et al. found that elevated AKI stage and SI serve as robust, independent predictors of in-hospital and 90-day mortality [30]. Furthermore, the integration of AKI stages with shock severity indices enhances mortality risk stratification compared to the use of either measure in isolation [30]. In this study, shock index was significantly better in 1000 and 2000 cc isotonic replacement, but unfortunately not indicative for competence of the treatment.

Pyakurel et al. describe that IVCCI is more accurate than PI for predicting fluid responsiveness [31]. Intravenous fluid resuscitation is a fundamental intervention in acute and critical care, particularly for conditions such as sepsis, trauma, and shock. The caval index, or percentage IVCC, serves as a predictor of preload reserve in addition to estimating central venous pressure (CVP). Muller et al. conducted a study on IVCC within an intensive care unit context characterized by diverse causes of circulatory failure [32]. The optimal IVCC cutoff for detecting volume responsiveness was determined to be 40%; however, this threshold failed to identify several fluid responders [32]. In this study, contrary to the findings of Pyakurel et al. [31], we could not find a significant difference between IVCCI and PI in evaluating fluid response in AKI patients. IVCCI is a static hemodynamic parameter, whereas PI is a dynamic one. We interpret that IVCCI and PI have comparable reliability in the assessment of fluid responsiveness.

PVI effectively estimates fluid responsiveness in critically ill and surgical patients undergoing mechanical ventilation [13,14]. To our knowledge, this study is the first to investigate PVI in non-intubated patients in the emergency department. Different from IVCC, MAP, SpO_2_, and SI, in addition to the meaningful follow-up data in the 1000 and 2000 cc groups, PVI shows a significant difference between 1000 and 2000 cc infusion. This shows the sensitivity of PVI to liquid loading dose. On the other hand, Savaşer et al. describe that intraoperative fluid management utilizing PVI monitoring enhances mean arterial pressure, oxygen saturation, lactate, and creatinine levels in thoracic surgery patients, while not significantly altering total fluid volume [33]. On the contrary, in a study by Monnet et al., they observed that PVI is an unreliable predictor of response to fluids in critically ill patients administered norepinephrine and may lack utility in certain patients [34]. Moreover, in a study by Broch et al., they found that the precision of PVI in forecasting fluid responsiveness is contingent upon the PI [35]. This indicates that in spontaneously breathing patients, PVI should be evaluated together with PI. In our study, although we created a statistical model combining PI and PVI, we did not reach statistical significance, which may be due to the limited number of patients. Further large-scale studies are needed to clarify the combined utility of PI and PVI.

Rather than other studies in the literature, in this study, we evaluate the PVI and mortality. The PVI level at admission (PVI-0) was found to be a significant predictor of mortality. On the other hand, the PVI after 1000 and 2000 cc isotonic resuscitation could not predict mortality. To our knowledge, this is also the first study to report an association between mortality and PVI in emergency department patients with AKI.

In this study, we demonstrated that the Pleth Variability Index (PVI) is a sensitive, non-invasive indicator of fluid responsiveness in non-intubated patients with acute kidney injury (AKI), and that baseline PVI values may have prognostic significance for mortality. These findings are consistent with those of Albayrak and Yüksel [36], who reported that PVI predicted 28-day mortality in intensive care patients with high sensitivity and specificity, and extend their observations to emergency department settings. Moreover, Liu et al. [37] showed in a meta-analysis that although the reliability of PVI varies across clinical contexts, it performs best in patients without surgery and in intensive care units—conditions comparable to those of our study population. By showing that PVI values decrease significantly in response to incremental isotonic fluid loading, even in spontaneously breathing patients, our results suggest that PVI can serve as a practical bedside alternative to ultrasound-based indices such as the Inferior Vena Cava Collapsibility Index (IVCCI), which are often operator-dependent. Collectively, these findings support incorporating PVI into early volume assessment and resuscitation strategies for AKI in the emergency department, while emphasizing the need for multicenter trials to validate its prognostic and hemodynamic utility.

### Limitations

This study has limitations. Small patient numbers and its nature as a single-center study limit generalizability. Most emergency department patients were admitted to wards, ICUs, or dialysis, so fluid resuscitation over 2000 mL and prolonged follow-up were impossible. This hindered advanced fluid responsiveness testing. PVI measures mechanically ventilated patients’ fluid responsiveness. However, this study was the first to use it on spontaneously breathing emergency department patients, which may raise measurement accuracy concerns. PVI values without mechanical ventilation may be unreliable due to irregular intrathoracic pressure changes. CVP, the gold standard for assessing hydration after fluid loading, was not used in this study due to its invasiveness and unsuitability for emergency departments. Thus, our parameters were not comparable to this gold standard. The operator, abdominal pressure, and patient positioning can affect ultrasonography IVCCI measurements, reducing their objectivity. Another limitation is that Doppler-based evaluations such as the Venous Excess Ultrasound (VExUS) score were not included, which could have provided additional information about venous congestion and fluid status. Since this was a prospective study without Doppler data collection, VExUS evaluation was not feasible; however, it is planned to be incorporated in future research. Future large-scale, multicenter, comparative studies should confirm our findings.

## 5. Conclusions

This study demonstrated that PVI is a more sensitive indicator of fluid responsiveness compared to other non-invasive parameters such as IVCCI, PI, MAP, SpO_2_, and SI, even in non-intubated patients with AKI. Moreover, PVI values measured at admission were found to have prognostic value in predicting patient outcomes. To better establish the clinical utility of PVI as a decision-support tool in emergency settings, these findings should be validated through larger, multicenter studies across diverse patient populations.

## Figures and Tables

**Table 1:** General Characteristics of the Patients.

Parameter	N	Minimum	Maximum	Mean	Std. Deviation
**Age**	50	19	91	69.68	15.15
**Systolic blood pressure**	50	40.00	184.00	114.3400	34.35
**Pulse**	50	72.00	170.00	101.5000	21.31
**Urea**	50	16.00	186.00	77.3400	40.33
**Creatinine**	50	1.70	13.14	4.10	2.47
**GFR**	50	2.78	37	16.89	9.23
**Lactate**	50	0.40	16.00	3.48	3.84
**pH**	50	6.84	7.52	7.30	0.15
**Na**	50	117.00	157.00	136.4600	8.11
**K**	50	2.76	7.80	4.86	1.09
**HCO_3_**	50	4.70	28.50	17.23	5.44

GFR: Glomerular filtration rate, HCO_3_: Bicarbonate.

**Table 2:** Means of pre-treatment, post-1000 cc and post-2000 cc isotonic infusion.

Treatment Status	Parameter	Minimum	Maximum	Mean	Std. Deviation
**Pre-treatment**	**MAP**	26.00	126.00	79.06	23.08
	**SpO_2_**	85.00	100.00	95.30	3.73
	**Pi**	0.07	7.20	1.66	1.98
	**PVI**	7.00	58.00	29.84	11.37
	**IVCCI**	0.50	0.83	0.52	0.19
	**SI**	0.45	4.14	1.04	0.66
**After 1000 cc of isotonic infusion**	**MAP**	37.00	120.00	82.52	18.07
	**SpO_2_**	85.00	100.00	96.18	2.97
	**Pi**	0.08	11.00	2.22	2.50
	**PVI**	4.00	53.00	24.48	11.13
	**IVCCI**	0.30	0.85	0.41	0.21
	**SI**	0.43	3.89	0.92	0.54
**After 2000 cc of isotonic infusion**	**MAP**	40.00	115.00	84.08	15.82
	**SpO_2_**	87.00	100.00	96.64	2.56
	**Pi**	0.14	16.00	2.52	3.20
	**PVI**	4.00	50.00	20.74	10.22
	**IVCCI**	0.40	0.85	0.39	0.18
	**SI**	0.05	3.46	0.80	0.48

MAP: mean arterial pressure, SpO_2_: oxygen saturation, Pi: perfusion index, PVI: Pleth Variability Index, IVCCI: Inferior Vena Cava Collapsibility Index, SI: Shock index.

**Table 3:** Comparison of the repeated measures of parameters before treatment, after 1000 cc isotonic infusion, and after 2000 cc isotonic infusion.

Repeated Parameters	Test Statistic	*p*
MAP	15.804	<0.001
SpO_2_	16.662	<0.001
PI	16.714	<0.001
PVI	61.797	<0.001
IVCCI	30.974	<0.001
SI	43.563	<0.001

MAP: mean arterial pressure, SpO_2_: oxygen saturation, PI: perfusion index, PVI: Pleth Variability Index, IVCCI: Inferior Vena Cava Collapsibility Index, SI: Shock index.

**Table 4:** Pairwise comparison of measurements obtained before treatment, after 1000 cc isotonic infusion, and after 2000 cc isotonic infusion.

	Sample 1 − Sample 2	Test Statistic	Std. Error	Std. Test Statistic	*p*
**MAP**	**T0 − T1**	−0.570	0.200	−2.850	**0.013**
	**T0 − T2**	−0.750	0.200	−3.750	**0.001**
	**T1 − T2**	−0.180	0.200	−0.900	1.000
**SpO_2_**	**T0 − T1**	−0.430	0.200	−2.150	0.095
	**T0 − T2**	−0.680	0.200	−3.400	**0.002**
	**T1 − T2**	−0.250	0.200	−1.250	0.634
**PI**	**T0 − T1**	−0.590	0.200	−2.950	**0.010**
	**T0 − T2**	−0.910	0.200	−4.550	**<0.001**
	**T1 − T2**	−0.320	0.200	−1.600	0.329
**PVI**	**T2 − T1**	0.700	0.200	3.500	**0.001**
	**T2 − T0**	1.610	0.200	8.050	**<0.001**
	**T1 − T0**	0.910	0.200	4.550	**<0.001**
**IVCCI**	**T2 − T1**	0.470	0.200	2.350	0.056
	**T2 − T0**	1.090	0.200	5.450	**<0.001**
	**T1 − T0**	0.620	0.200	3.100	**0.006**
**SI**	**T2 − T1**	0.310	0.200	1.550	0.363
	**T2 − T0**	0.860	0.200	4.300	**<0.001**
	**T1 − T0**	0.550	0.200	2.750	**0.018**

MAP: mean arterial pressure, SpO_2_ oxygen saturation, Pi: perfusion index, PVI: Pleth Variability Index, IVCCI: Inferior Vena Cava Collapsibility Index, SI: Shock index, T0: pre-treatment, T1: after 1000 cc of isotonic infusion, T2: after 2000 cc of isotonic infusion.

**Table 5:** Comparison of PVI values for mortality prediction.

Parameter	Mortality	N	Mean	Std. Deviation	Std. Error Mean	*p*
**PVI-0**	Survive	42	28.8810	11.69157	1.80405	**<0.001**
	Ex	8	47.2500	17.92644	6.33795	
**PVI-1**	Survive	42	24.3095	11.32338	1.74724	0.529
	Ex	8	27.0000	8.91227	3.15096	
**PVI-2**	Survive	42	20.8095	10.49562	1.61951	0.914
	Ex	8	20.3750	9.33408	3.30009	

PVI-0: baseline Pleth Variability Index, PVI-1: Pleth Variability Index after 1000 cc of isotonic infusion, PVI-2: Pleth Variability Index after 2000 cc of isotonic infusion.

**Table 6:** The success of pre-treatment PVI in predicting mortality.

Parameter	Optimal Cut-Off	Youden Index	Sensitivity	Spesifity	PPV	NPV
**PVI-0**	**36.5**	**0.637**	**0.875**	**0.762**	0.412	**0.970**

## Data Availability

Anonymized data supporting this study’s findings are available from the corresponding author upon reasonable request.

## Abbreviations

The following abbreviations are used in this manuscript:

AKI	Acute Kidney Injury
IVC	Inferior Vena Cava
IVCCI	Inferior Vena Cava Collapsibility Index
PI	Perfusion Index
PVI	Pleth Variability Index
SI	Shock Index
MAP	Mean Arterial Pressure
CVP	Central Venous Pressure
SpO_2_	Peripheral Oxygen Saturation
KDIGO	Kidney Disease: Improving Global Outcomes
